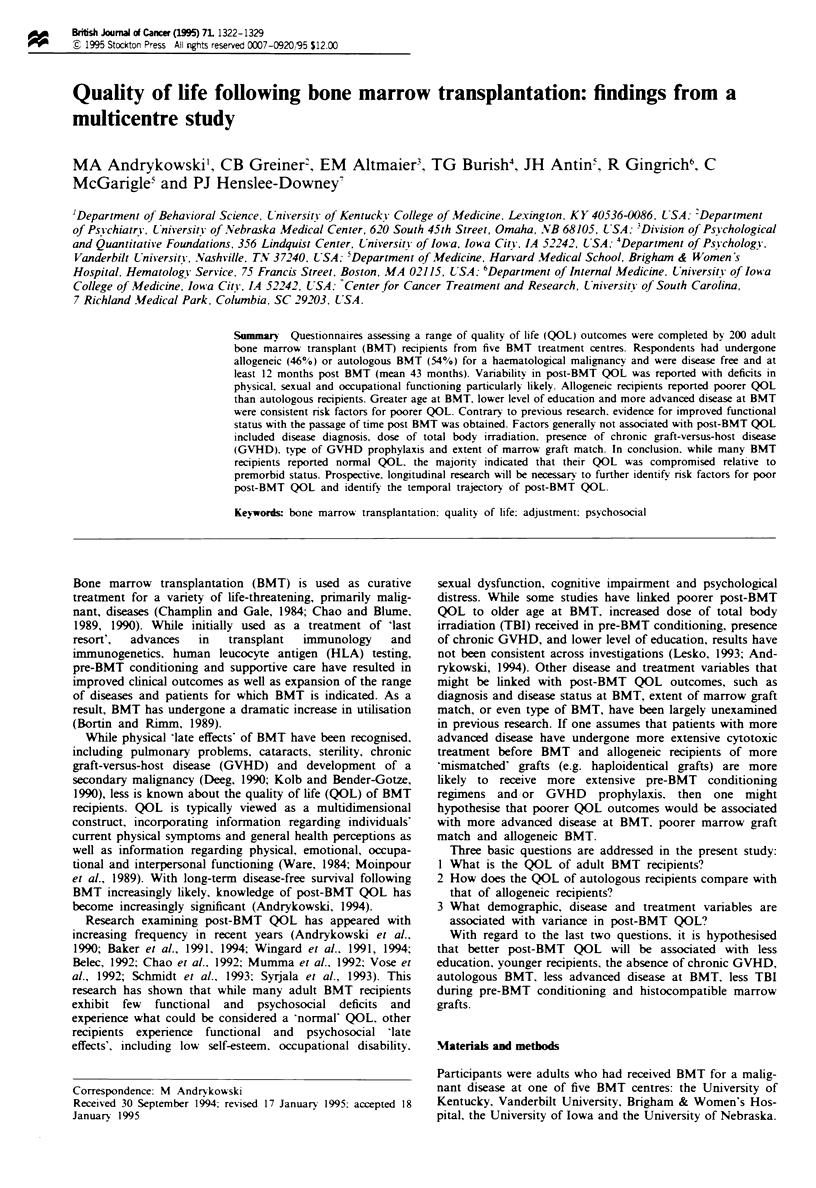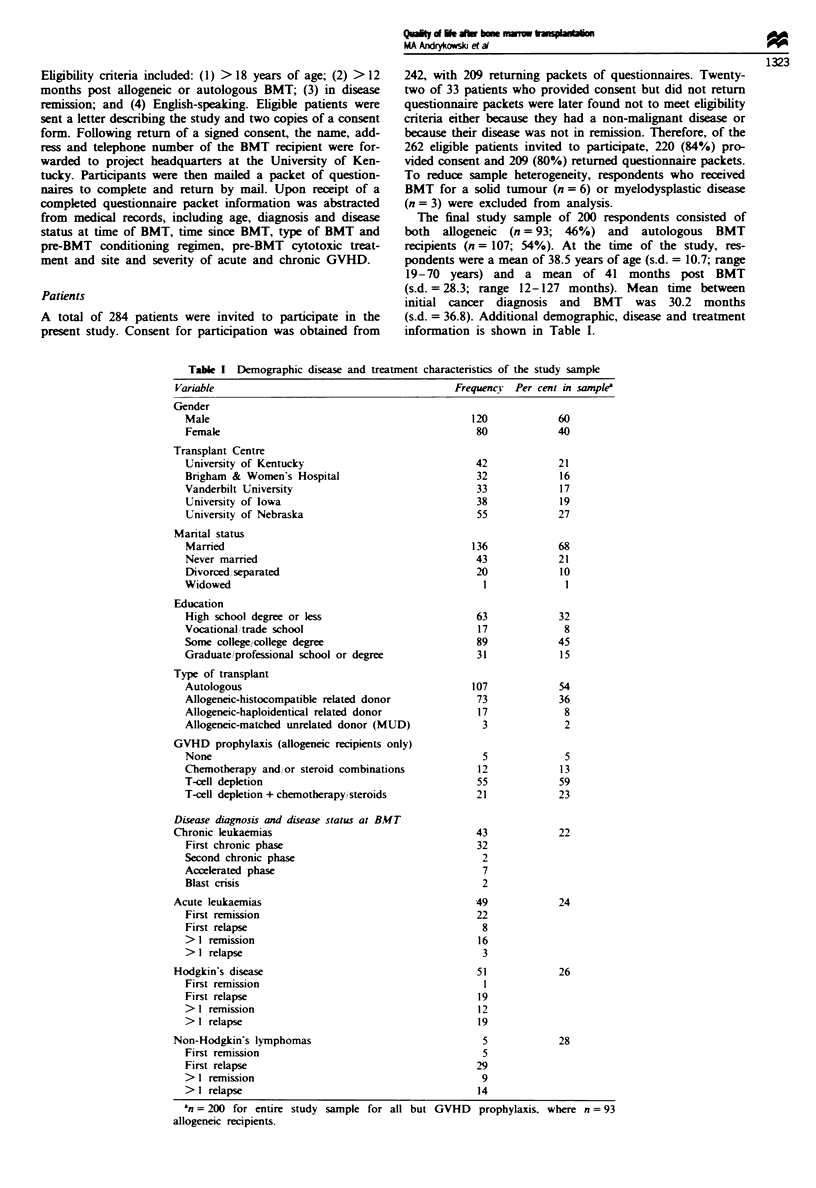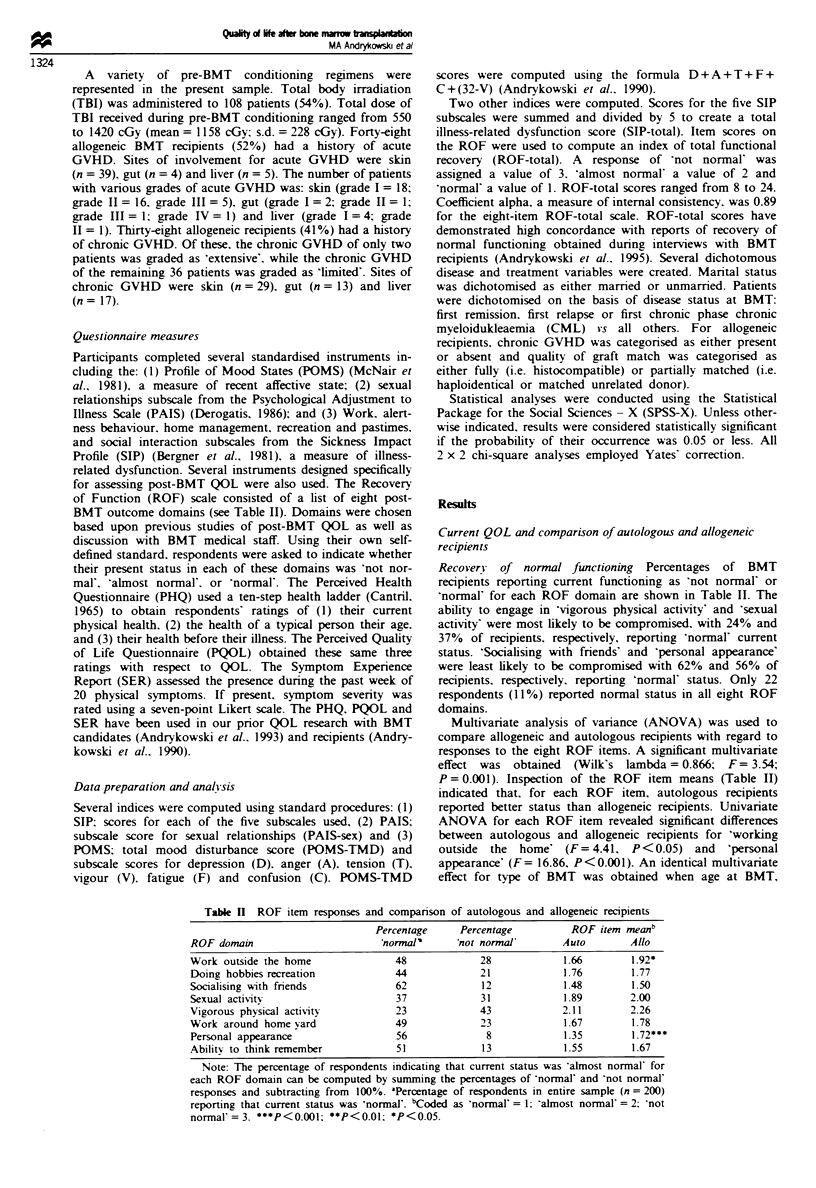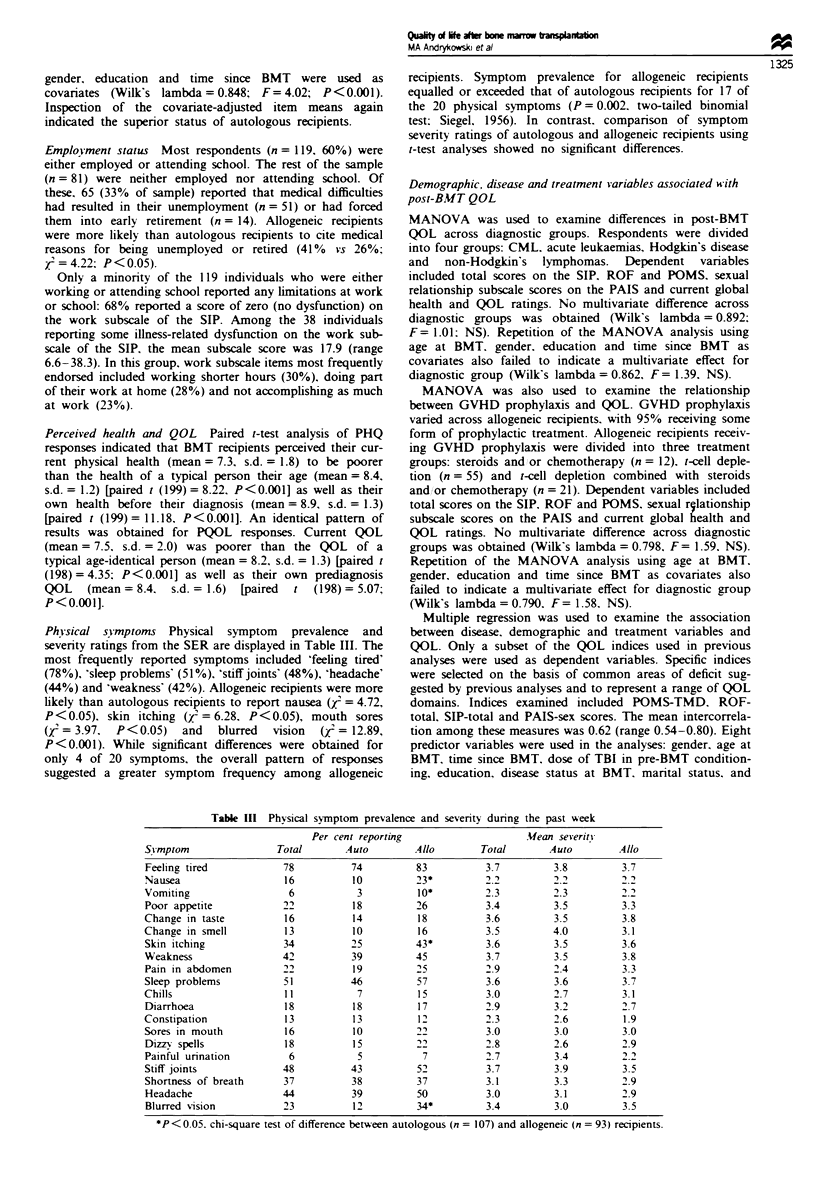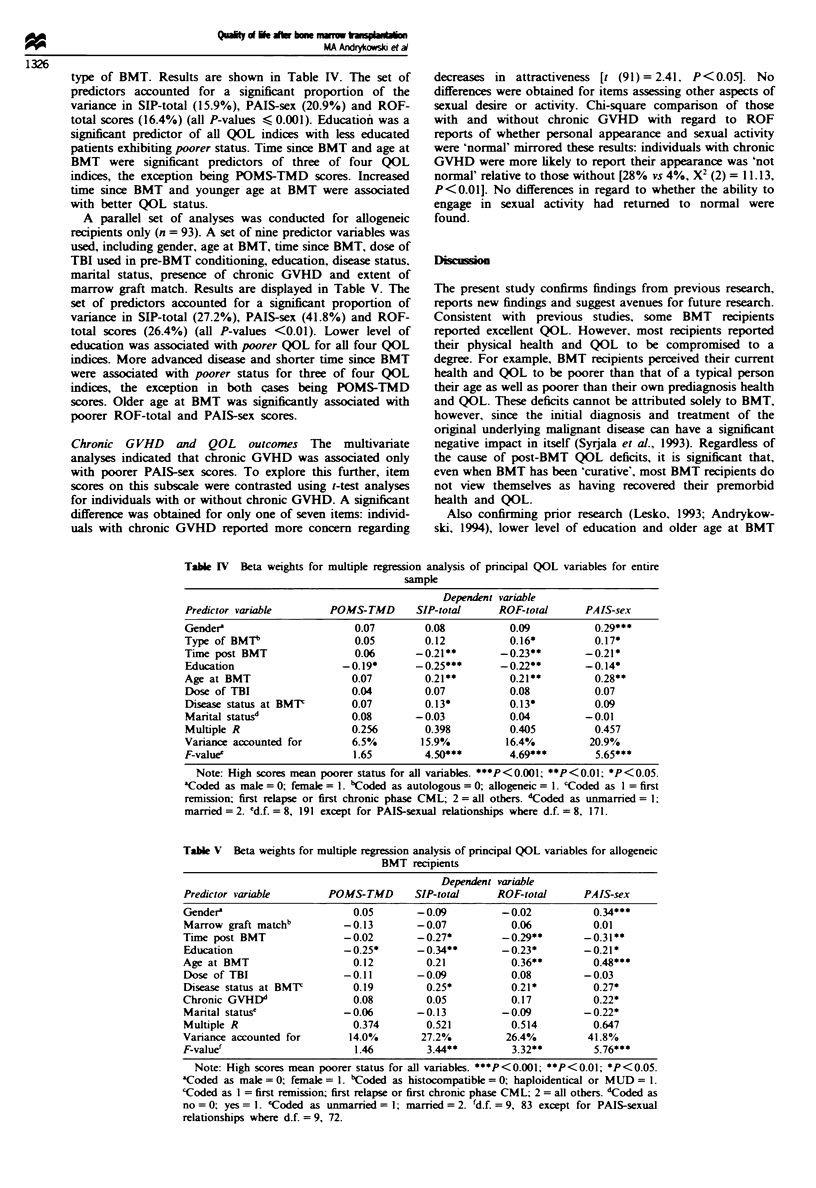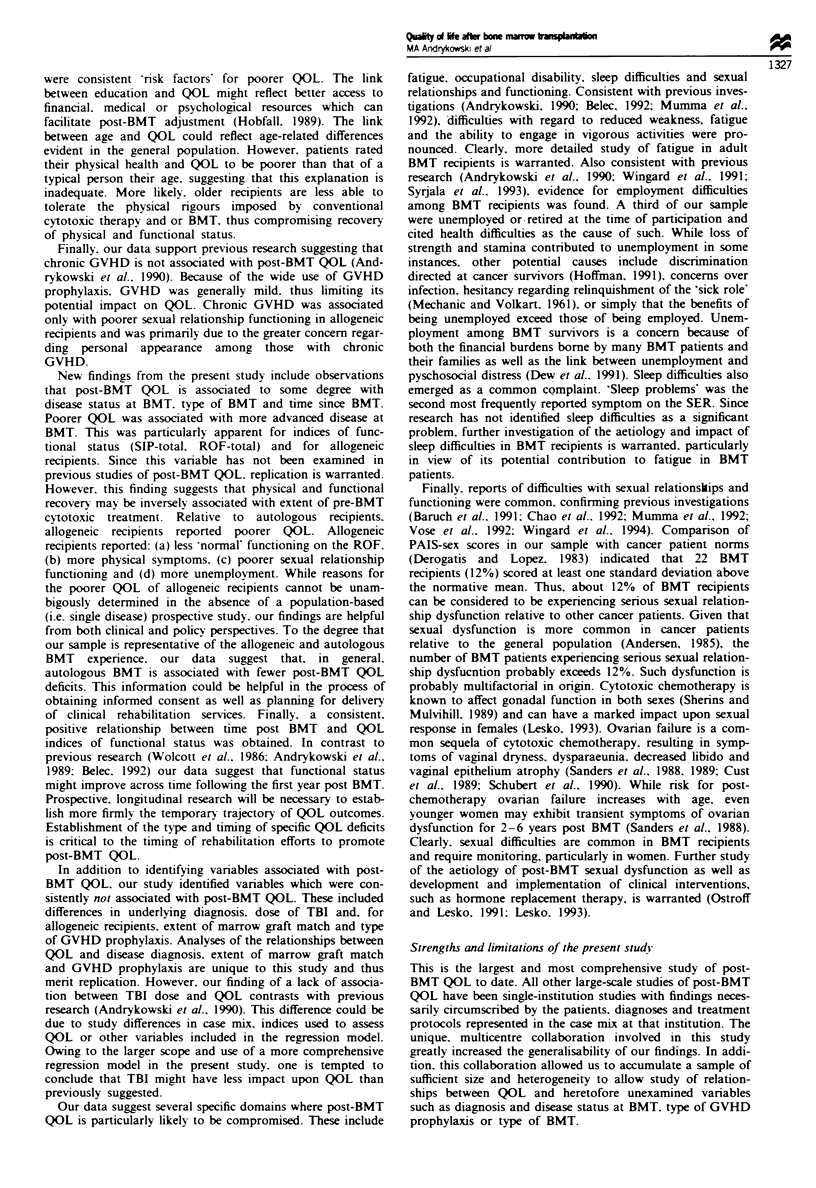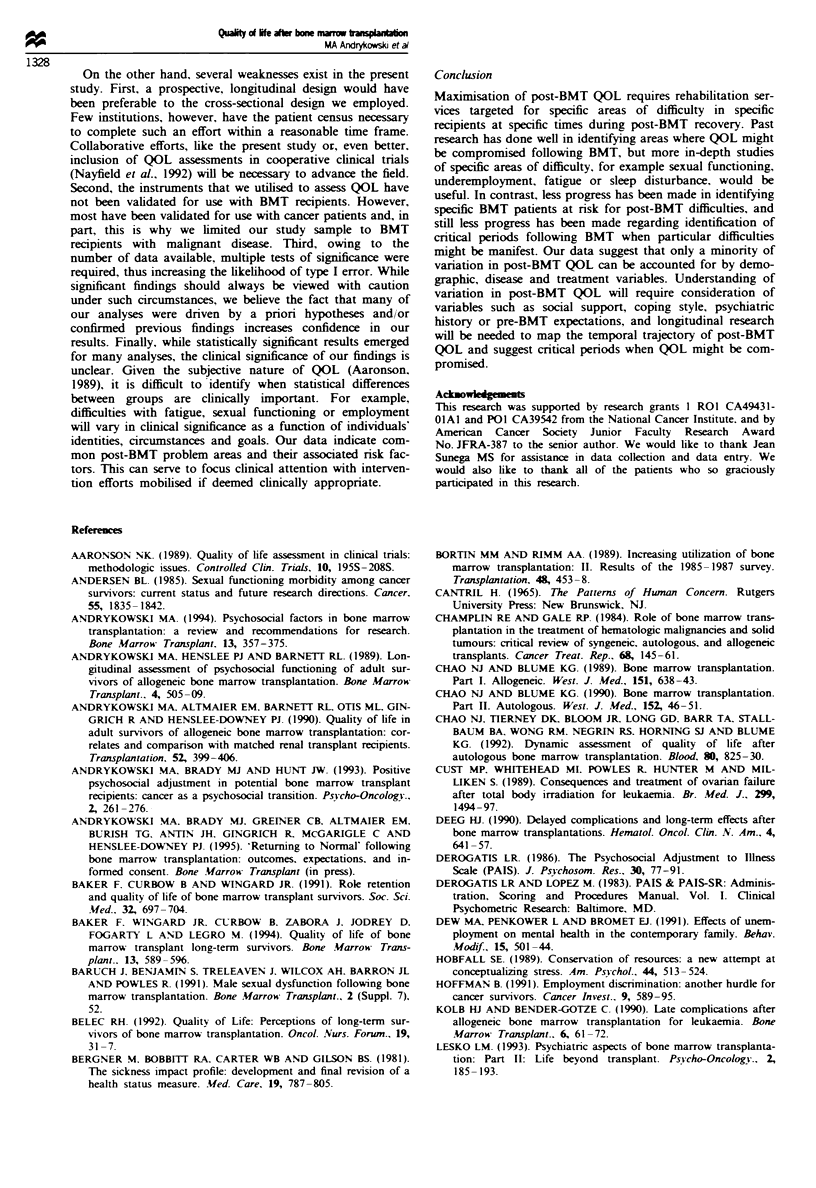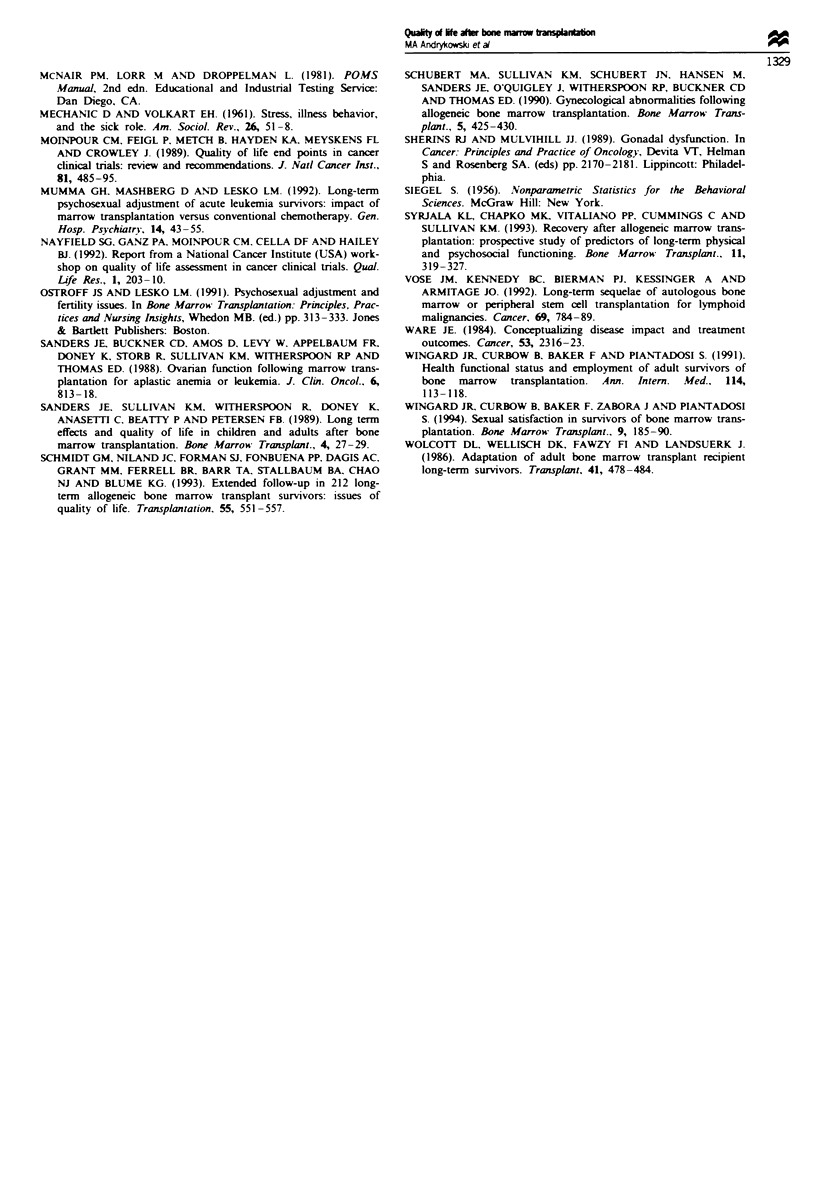# Quality of life following bone marrow transplantation: findings from a multicentre study.

**DOI:** 10.1038/bjc.1995.257

**Published:** 1995-06

**Authors:** M. A. Andrykowski, C. B. Greiner, E. M. Altmaier, T. G. Burish, J. H. Antin, R. Gingrich, C. McGarigle, P. J. Henslee-Downey

**Affiliations:** Department of Behavioral Science, University of Kentucky College of Medicine, Lexington 40536-0086, USA.

## Abstract

Questionnaires assessing a range of quality of life (QOL) outcomes were completed by 200 adult bone marrow transplant (BMT) recipients from five BMT treatment centres. Respondents had undergone allogeneic (46%) or autologous BMT (54%) for a haematological malignancy and were disease free and at least 12 months post BMT (mean 43 months). Variability in post-BMT QOL was reported with deficits in physical, sexual and occupational functioning particularly likely. Allogeneic recipients reported poorer QOL than autologous recipients. Greater age at BMT, lower level of education and more advanced disease at BMT were consistent risk factors for poorer QOL. Contrary to previous research, evidence for improved functional status with the passage of time post BMT was obtained. Factors generally not associated with post-BMT QOL included disease diagnosis, dose of total body irradiation, presence of chronic graft-versus-host disease (GVHD), type of GVHD prophylaxis and extent of marrow graft match. In conclusion, while many BMT recipients reported normal QOL, the majority indicated that their QOL was compromised relative to premorbid status. Prospective, longitudinal research will be necessary to further identify risk factors for poor post-BMT QOL and identify the temporal trajectory of post-BMT QOL.


British Journal of Cancer (1995) 71, 1322-1329

© 1995 Stockton Press All rights reserved 0007-0920/95 $12.00

### Quality of life following bone marrow transplantation: findings from a multicentre study

MA Andrykowski[1], CB Greiner[2], EM Altmaier[3], TG Burish[4], JH Antin[5], R Gingrich[6], C McGarigle[5] and PJ Henslee-Downey[7]

*[1]Department of Behavioral Science, University of Kentucky College of Medicine, Lexington, KY 40536-0086, USA; [2]Department of Psychiatry, University of Nebraska Medical Center, 620 South 45th Street, Omaha, NB 68105, USA; [3]Division of Psychological and Quantitative Foundations, 356 Lindquist Center, University of Iowa, Iowa City, IA 52242, USA; [4]Department of Psychology, Vanderbilt University, Nashville, TN 37240, USA; [5]Department of Medicine, Harvard Medical School, Brigham & Women's Hospital, Hematology Service, 75 Francis Street, Boston, MA 02115, USA; [6]Department of Internal Medicine, University of Iowa College of Medicine, Iowa City, IA 52242, USA; [7]Center for Cancer Treatment and Research, University of South Carolina, 7 Richland Medical Park, Columbia, SC 29203, USA.*

**Summary** Questionnaires assessing a range of quality of life (QOL) outcomes were completed by 200 adult bone marrow transplant (BMT) recipients from five BMT treatment centres. Respondents had undergone allogeneic (46%) or autologous BMT (54%) for a haematological malignancy and were disease free and at least 12 months post BMT (mean 43 months). Variability in post-BMT QOL was reported with deficits in physical, sexual and occupational functioning particularly likely. Allogeneic recipients reported poorer QOL than autologous recipients. Greater age at BMT, lower level of education and more advanced disease at BMT were consistent risk factors for poorer QOL. Contrary to previous research, evidence for improved functional status with the passage of time post BMT was obtained. Factors generally not associated with post-BMT QOL included disease diagnosis, dose of total body irradiation, presence of chronic graft-versus-host disease (GVHD), type of GVHD prophylaxis and extent of marrow graft match. In conclusion, while many BMT recipients reported normal QOL, the majority indicated that their QOL was compromised relative to premorbid status. Prospective, longitudinal research will be necessary to further identify risk factors for poor post-BMT QOL and identify the temporal trajectory of post-BMT QOL.

**Keywords:** bone marrow transplantation; quality of life; adjustment; psychosocial

Bone marrow transplantation (BMT) is used as curative treatment for a variety of life-threatening, primarily malignant, diseases (Champlin and Gale, 1984; Chao and Blume, 1989, 1990). While initially used as a treatment of 'last resort', advances in transplant immunology and immunogenetics, human leucocyte antigen (HLA) testing, pre-BMT conditioning and supportive care have resulted in improved clinical outcomes as well as expansion of the range of diseases and patients for which BMT is indicated. As a result, BMT has undergone a dramatic increase in utilisation (Bortin and Rimm, 1989).

While physical 'late effects' of BMT have been recognised, including pulmonary problems, cataracts, sterility, chronic graft-versus-host disease (GVHD) and development of a secondary malignancy (Deeg, 1990; Kolb and Bender-Gotze, 1990), less is known about the quality of life (QOL) of BMT recipients. QOL is typically viewed as a multidimensional construct, incorporating information regarding individuals' current physical symptoms and general health perceptions as well as information regarding physical, emotional, occupational and interpersonal functioning (Ware, 1984; Moinpour *et al.*, 1989). With long-term disease-free survival following BMT increasingly likely, knowledge of post-BMT QOL has become increasingly significant (Andrykowski, 1994).

Research examining post-BMT QOL has appeared with increasing frequency in recent years (Andrykowski *et al.*, 1990; Baker *et al.*, 1991, 1994; Wingard *et al.*, 1991, 1994; Belec, 1992; Chao *et al.*, 1992; Mumma *et al.*, 1992; Vose *et al.*, 1992; Schmidt *et al.*, 1993; Syrjala *et al.*, 1993). This research has shown that while many adult BMT recipients exhibit few functional and psychosocial deficits and experience what could be considered a 'normal' QOL, other recipients experience functional and psychosocial 'late effects', including low self-esteem, occupational disability, sexual dysfunction, cognitive impairment and psychological distress. While some studies have linked poorer post-BMT QOL to older age at BMT, increased dose of total body irradiation (TBI) received in pre-BMT conditioning, presence of chronic GVHD, and lower level of education, results have not been consistent across investigations (Lesko, 1993; Andrykowski, 1994). Other disease and treatment variables that might be linked with post-BMT QOL outcomes, such as diagnosis and disease status at BMT, extent of marrow graft match, or even type of BMT, have been largely unexamined in previous research. If one assumes that patients with more advanced disease have undergone more extensive cytotoxic treatment before BMT and allogeneic recipients of more 'mismatched' grafts (e.g. haploidentical grafts) are more likely to receive more extensive pre-BMT conditioning regimens and/or GVHD prophylaxis, then one might hypothesise that poorer QOL outcomes would be associated with more advanced disease at BMT, poorer marrow graft match and allogeneic BMT.

Three basic questions are addressed in the present study:

1 What is the QOL of adult BMT recipients?
2 How does the QOL of autologous recipients compare with that of allogeneic recipients?
3 What demographic, disease and treatment variables are associated with variance in post-BMT QOL?

With regard to the last two questions, it is hypothesised that better post-BMT QOL will be associated with less education, younger recipients, the absence of chronic GVHD, autologous BMT, less advanced disease at BMT, less TBI during pre-BMT conditioning and histocompatible marrow grafts.

### Materials and methods

Participants were adults who had received BMT for a malignant disease at one of five BMT centres: the University of Kentucky, Vanderbilt University, Brigham & Women's Hospital, the University of Iowa and the University of Nebraska.

Correspondence: M Andrykowski
Received 30 September 1994; revised 17 January 1995; accepted 18 January 1995

Eligibility criteria included: (1) >18 years of age; (2) >12 months post allogeneic or autologous BMT; (3) in disease remission; and (4) English-speaking. Eligible patients were sent a letter describing the study and two copies of a consent form. Following return of a signed consent, the name, address and telephone number of the BMT recipient were forwarded to project headquarters at the University of Kentucky. Participants were then mailed a packet of questionnaires to complete and return by mail. Upon receipt of a completed questionnaire packet information was abstracted from medical records, including age, diagnosis and disease status at time of BMT, time since BMT, type of BMT and pre-BMT conditioning regimen, pre-BMT cytotoxic treatment and site and severity of acute and chronic GVHD.

### Patients

A total of 284 patients were invited to participate in the present study. Consent for participation was obtained from 242, with 209 returning packets of questionnaires. Twenty-two of 33 patients who provided consent but did not return questionnaire packets were later found not to meet eligibility criteria either because they had a non-malignant disease or because their disease was not in remission. Therefore, of the 262 eligible patients invited to participate, 220 (84%) provided consent and 209 (80%) returned questionnaire packets. To reduce sample heterogeneity, respondents who received BMT for a solid tumour ($n = 6$) or myelodysplastic disease ($n = 3$) were excluded from analysis.

The final study sample of 200 respondents consisted of both allogeneic ($n = 93$; 46%) and autologous BMT recipients ($n = 107$; 54%). At the time of the study, respondents were a mean of 38.5 years of age (s.d. = 10.7; range 19–70 years) and a mean of 41 months post BMT (s.d. = 28.3; range 12–127 months). Mean time between initial cancer diagnosis and BMT was 30.2 months (s.d. = 36.8). Additional demographic, disease and treatment information is shown in Table I.

**Table I** Demographic disease and treatment characteristics of the study sample

| *Variable* | *Frequency* | *Per cent in sample*[a] |
|---|---|---|
| Gender | | |
| Male | 120 | 60 |
| Female | 80 | 40 |
| Transplant Centre | | |
| University of Kentucky | 42 | 21 |
| Brigham & Women's Hospital | 32 | 16 |
| Vanderbilt University | 33 | 17 |
| University of Iowa | 38 | 19 |
| University of Nebraska | 55 | 27 |
| Marital status | | |
| Married | 136 | 68 |
| Never married | 43 | 21 |
| Divorced/separated | 20 | 10 |
| Widowed | 1 | 1 |
| Education | | |
| High school degree or less | 63 | 32 |
| Vocational/trade school | 17 | 8 |
| Some college/college degree | 89 | 45 |
| Graduate/professional school or degree | 31 | 15 |
| Type of transplant | | |
| Autologous | 107 | 54 |
| Allogeneic-histocompatible related donor | 73 | 36 |
| Allogeneic-haploidentical related donor | 17 | 8 |
| Allogeneic-matched unrelated donor (MUD) | 3 | 2 |
| GVHD prophylaxis (allogeneic recipients only) | | |
| None | 5 | 5 |
| Chemotherapy and/or steroid combinations | 12 | 13 |
| T-cell depletion | 55 | 59 |
| T-cell depletion + chemotherapy/steroids | 21 | 23 |
| *Disease diagnosis and disease status at BMT* | | |
| Chronic leukaemias | 43 | 22 |
| First chronic phase | 32 | |
| Second chronic phase | 2 | |
| Accelerated phase | 7 | |
| Blast crisis | 2 | |
| Acute leukaemias | 49 | 24 |
| First remission | 22 | |
| First relapse | 8 | |
| >1 remission | 16 | |
| >1 relapse | 3 | |
| Hodgkin's disease | 51 | 26 |
| First remission | 1 | |
| First relapse | 19 | |
| >1 remission | 12 | |
| >1 relapse | 19 | |
| Non-Hodgkin's lymphomas | 5 | 28 |
| First remission | 5 | |
| First relapse | 29 | |
| >1 remission | 9 | |
| >1 relapse | 14 | |

[a] $n = 200$ for entire study sample for all but GVHD prophylaxis, where $n = 93$ allogeneic recipients.

A variety of pre-BMT conditioning regimens were represented in the present sample. Total body irradiation (TBI) was administered to 108 patients (54%). Total dose of TBI received during pre-BMT conditioning ranged from 550 to 1420 cGy (mean = 1158 cGy; s.d. = 228 cGy). Forty-eight allogeneic BMT recipients (52%) had a history of acute GVHD. Sites of involvement for acute GVHD were skin ($n = 39$), gut ($n = 4$) and liver ($n = 5$). The number of patients with various grades of acute GVHD was: skin (grade I = 18; grade II = 16, grade III = 5), gut (grade I = 2; grade II = 1; grade III = 1; grade IV = 1) and liver (grade I = 4; grade II = 1). Thirty-eight allogeneic recipients (41%) had a history of chronic GVHD. Of these, the chronic GVHD of only two patients was graded as 'extensive', while the chronic GVHD of the remaining 36 patients was graded as 'limited'. Sites of chronic GVHD were skin ($n = 29$), gut ($n = 13$) and liver ($n = 17$).

### *Questionnaire measures*

Participants completed several standardised instruments including the: (1) Profile of Mood States (POMS) (McNair *et al.*, 1981), a measure of recent affective state; (2) sexual relationships subscale from the Psychological Adjustment to Illness Scale (PAIS) (Derogatis, 1986); and (3) Work, alertness behaviour, home management, recreation and pastimes, and social interaction subscales from the Sickness Impact Profile (SIP) (Bergner *et al.*, 1981), a measure of illness-related dysfunction. Several instruments designed specifically for assessing post-BMT QOL were also used. The Recovery of Function (ROF) scale consisted of a list of eight post-BMT outcome domains (see Table II). Domains were chosen based upon previous studies of post-BMT QOL as well as discussion with BMT medical staff. Using their own self-defined standard, respondents were asked to indicate whether their present status in each of these domains was 'not normal', 'almost normal', or 'normal'. The Perceived Health Questionnaire (PHQ) used a ten-step health ladder (Cantril, 1965) to obtain respondents' ratings of (1) their current physical health, (2) the health of a typical person their age, and (3) their health before their illness. The Perceived Quality of Life Questionnaire (PQOL) obtained these same three ratings with respect to QOL. The Symptom Experience Report (SER) assessed the presence during the past week of 20 physical symptoms. If present, symptom severity was rated using a seven-point Likert scale. The PHQ, PQOL and SER have been used in our prior QOL research with BMT candidates (Andrykowski *et al.*, 1993) and recipients (Andrykowski *et al.*, 1990).

### *Data preparation and analysis*

Several indices were computed using standard procedures: (1) SIP; scores for each of the five subscales used, (2) PAIS; subscale score for sexual relationships (PAIS-sex) and (3) POMS; total mood disturbance score (POMS-TMD) and subscale scores for depression (D), anger (A), tension (T), vigour (V), fatigue (F) and confusion (C). POMS-TMD scores were computed using the formula $D+A+T+F+C+(32-V)$ (Andrykowski *et al.*, 1990).

Two other indices were computed. Scores for the five SIP subscales were summed and divided by 5 to create a total illness-related dysfunction score (SIP-total). Item scores on the ROF were used to compute an index of total functional recovery (ROF-total). A response of 'not normal' was assigned a value of 3, 'almost normal' a value of 2 and 'normal' a value of 1. ROF-total scores ranged from 8 to 24. Coefficient alpha, a measure of internal consistency, was 0.89 for the eight-item ROF-total scale. ROF-total scores have demonstrated high concordance with reports of recovery of normal functioning obtained during interviews with BMT recipients (Andrykowski *et al.*, 1995). Several dichotomous disease and treatment variables were created. Marital status was dichotomised as either married or unmarried. Patients were dichotomised on the basis of disease status at BMT: first remission, first relapse or first chronic phase chronic myeloidukleaemia (CML) *vs* all others. For allogeneic recipients, chronic GVHD was categorised as either present or absent and quality of graft match was categorised as either fully (i.e. histocompatible) or partially matched (i.e. haploidentical or matched unrelated donor).

Statistical analyses were conducted using the Statistical Package for the Social Sciences – X (SPSS-X). Unless otherwise indicated, results were considered statistically significant if the probability of their occurrence was 0.05 or less. All 2 × 2 chi-square analyses employed Yates' correction.

### Results

### *Current QOL and comparison of autologous and allogeneic recipients*

*Recovery of normal functioning* Percentages of BMT recipients reporting current functioning as 'not normal' or 'normal' for each ROF domain are shown in Table II. The ability to engage in 'vigorous physical activity' and 'sexual activity' were most likely to be compromised, with 24% and 37% of recipients, respectively, reporting 'normal' current status. 'Socialising with friends' and 'personal appearance' were least likely to be compromised with 62% and 56% of recipients, respectively, reporting 'normal' status. Only 22 respondents (11%) reported normal status in all eight ROF domains.

Multivariate analysis of variance (ANOVA) was used to compare allogeneic and autologous recipients with regard to responses to the eight ROF items. A significant multivariate effect was obtained (Wilk's lambda = 0.866; $F = 3.54$; $P = 0.001$). Inspection of the ROF item means (Table II) indicated that, for each ROF item, autologous recipients reported better status than allogeneic recipients. Univariate ANOVA for each ROF item revealed significant differences between autologous and allogeneic recipients for 'working outside the home' ($F = 4.41$, $P < 0.05$) and 'personal appearance' ($F = 16.86$, $P < 0.001$). An identical multivariate effect for type of BMT was obtained when age at BMT,

**Table II** ROF item responses and comparison of autologous and allogeneic recipients

| *ROF domain* | *Percentage 'normal'*[a] | *Percentage 'not normal'* | *ROF item mean*[b] *Auto* | *Allo* |
|---|---|---|---|---|
| Work outside the home | 48 | 28 | 1.66 | 1.92* |
| Doing hobbies recreation | 44 | 21 | 1.76 | 1.77 |
| Socialising with friends | 62 | 12 | 1.48 | 1.50 |
| Sexual activity | 37 | 31 | 1.89 | 2.00 |
| Vigorous physical activity | 23 | 43 | 2.11 | 2.26 |
| Work around home yard | 49 | 23 | 1.67 | 1.78 |
| Personal appearance | 56 | 8 | 1.35 | 1.72*** |
| Ability to think remember | 51 | 13 | 1.55 | 1.67 |

Note: The percentage of respondents indicating that current status was 'almost normal' for each ROF domain can be computed by summing the percentages of 'normal' and 'not normal' responses and subtracting from 100%. [a]Percentage of respondents in entire sample ($n = 200$) reporting that current status was 'normal'. [b]Coded as 'normal' = 1; 'almost normal' = 2; 'not normal' = 3. ***$P < 0.001$; **$P < 0.01$; *$P < 0.05$.

gender, education and time since BMT were used as covariates (Wilk's lambda = 0.848; $F = 4.02$; $P < 0.001$). Inspection of the covariate-adjusted item means again indicated the superior status of autologous recipients.

*Employment status* Most respondents ($n = 119$, 60%) were either employed or attending school. The rest of the sample ($n = 81$) were neither employed nor attending school. Of these, 65 (33% of sample) reported that medical difficulties had resulted in their unemployment ($n = 51$) or had forced them into early retirement ($n = 14$). Allogeneic recipients were more likely than autologous recipients to cite medical reasons for being unemployed or retired (41% *vs* 26%; $\chi^2 = 4.22$; $P < 0.05$).

Only a minority of the 119 individuals who were either working or attending school reported any limitations at work or school: 68% reported a score of zero (no dysfunction) on the work subscale of the SIP. Among the 38 individuals reporting some illness-related dysfunction on the work subscale of the SIP, the mean subscale score was 17.9 (range 6.6–38.3). In this group, work subscale items most frequently endorsed included working shorter hours (30%), doing part of their work at home (28%) and not accomplishing as much at work (23%).

*Perceived health and QOL* Paired *t*-test analysis of PHQ responses indicated that BMT recipients perceived their current physical health (mean = 7.3, s.d. = 1.8) to be poorer than the health of a typical person their age (mean = 8.4, s.d. = 1.2) [paired $t$ (199) = 8.22, $P < 0.001$] as well as their own health before their diagnosis (mean = 8.9, s.d. = 1.3) [paired $t$ (199) = 11.18, $P < 0.001$]. An identical pattern of results was obtained for PQOL responses. Current QOL (mean = 7.5, s.d. = 2.0) was poorer than the QOL of a typical age-identical person (mean = 8.2, s.d. = 1.3) [paired $t$ (198) = 4.35; $P < 0.001$] as well as their own prediagnosis QOL (mean = 8.4, s.d. = 1.6) [paired $t$ (198) = 5.07; $P < 0.001$].

*Physical symptoms* Physical symptom prevalence and severity ratings from the SER are displayed in Table III. The most frequently reported symptoms included 'feeling tired' (78%), 'sleep problems' (51%), 'stiff joints' (48%), 'headache' (44%) and 'weakness' (42%). Allogeneic recipients were more likely than autologous recipients to report nausea ($\chi^2 = 4.72$, $P < 0.05$), skin itching ($\chi^2 = 6.28$, $P < 0.05$), mouth sores ($\chi^2 = 3.97$, $P < 0.05$) and blurred vision ($\chi^2 = 12.89$, $P < 0.001$). While significant differences were obtained for only 4 of 20 symptoms, the overall pattern of responses suggested a greater symptom frequency among allogeneic recipients. Symptom prevalence for allogeneic recipients equalled or exceeded that of autologous recipients for 17 of the 20 physical symptoms ($P = 0.002$, two-tailed binomial test; Siegel, 1956). In contrast, comparison of symptom severity ratings of autologous and allogeneic recipients using *t*-test analyses showed no significant differences.

*Demographic, disease and treatment variables associated with post-BMT QOL*

MANOVA was used to examine differences in post-BMT QOL across diagnostic groups. Respondents were divided into four groups: CML, acute leukaemias, Hodgkin's disease and non-Hodgkin's lymphomas. Dependent variables included total scores on the SIP, ROF and POMS, sexual relationship subscale scores on the PAIS and current global health and QOL ratings. No multivariate difference across diagnostic groups was obtained (Wilk's lambda = 0.892; $F = 1.01$; NS). Repetition of the MANOVA analysis using age at BMT, gender, education and time since BMT as covariates also failed to indicate a multivariate effect for diagnostic group (Wilk's lambda = 0.862, $F = 1.39$, NS).

MANOVA was also used to examine the relationship between GVHD prophylaxis and QOL. GVHD prophylaxis varied across allogeneic recipients, with 95% receiving some form of prophylactic treatment. Allogeneic recipients receiving GVHD prophylaxis were divided into three treatment groups: steroids and/or chemotherapy ($n = 12$), *t*-cell depletion ($n = 55$) and *t*-cell depletion combined with steroids and/or chemotherapy ($n = 21$). Dependent variables included total scores on the SIP, ROF and POMS, sexual relationship subscale scores on the PAIS and current global health and QOL ratings. No multivariate difference across diagnostic groups was obtained (Wilk's lambda = 0.798, $F = 1.59$, NS). Repetition of the MANOVA analysis using age at BMT, gender, education and time since BMT as covariates also failed to indicate a multivariate effect for diagnostic group (Wilk's lambda = 0.790, $F = 1.58$, NS).

Multiple regression was used to examine the association between disease, demographic and treatment variables and QOL. Only a subset of the QOL indices used in previous analyses were used as dependent variables. Specific indices were selected on the basis of common areas of deficit suggested by previous analyses and to represent a range of QOL domains. Indices examined included POMS-TMD, ROF-total, SIP-total and PAIS-sex scores. The mean intercorrelation among these measures was 0.62 (range 0.54–0.80). Eight predictor variables were used in the analyses: gender, age at BMT, time since BMT, dose of TBI in pre-BMT conditioning, education, disease status at BMT, marital status, and

**Table III** Physical symptom prevalence and severity during the past week

| | *Per cent reporting* | | | *Mean severity* | | |
|---|---|---|---|---|---|---|
| *Symptom* | *Total* | *Auto* | *Allo* | *Total* | *Auto* | *Allo* |
| Feeling tired | 78 | 74 | 83 | 3.7 | 3.8 | 3.7 |
| Nausea | 16 | 10 | 23* | 2.2 | 2.2 | 2.2 |
| Vomiting | 6 | 3 | 10* | 2.3 | 2.3 | 2.2 |
| Poor appetite | 22 | 18 | 26 | 3.4 | 3.5 | 3.3 |
| Change in taste | 16 | 14 | 18 | 3.6 | 3.5 | 3.8 |
| Change in smell | 13 | 10 | 16 | 3.5 | 4.0 | 3.1 |
| Skin itching | 34 | 25 | 43* | 3.6 | 3.5 | 3.6 |
| Weakness | 42 | 39 | 45 | 3.7 | 3.5 | 3.8 |
| Pain in abdomen | 22 | 19 | 25 | 2.9 | 2.4 | 3.3 |
| Sleep problems | 51 | 46 | 57 | 3.6 | 3.6 | 3.7 |
| Chills | 11 | 7 | 15 | 3.0 | 2.7 | 3.1 |
| Diarrhoea | 18 | 18 | 17 | 2.9 | 3.2 | 2.7 |
| Constipation | 13 | 13 | 12 | 2.3 | 2.6 | 1.9 |
| Sores in mouth | 16 | 10 | 22 | 3.0 | 3.0 | 3.0 |
| Dizzy spells | 18 | 15 | 22 | 2.8 | 2.6 | 2.9 |
| Painful urination | 6 | 5 | 7 | 2.7 | 3.4 | 2.2 |
| Stiff joints | 48 | 43 | 52 | 3.7 | 3.9 | 3.5 |
| Shortness of breath | 37 | 38 | 37 | 3.1 | 3.3 | 2.9 |
| Headache | 44 | 39 | 50 | 3.0 | 3.1 | 2.9 |
| Blurred vision | 23 | 12 | 34* | 3.4 | 3.0 | 3.5 |

*$P < 0.05$, chi-square test of difference between autologous ($n = 107$) and allogeneic ($n = 93$) recipients.

type of BMT. Results are shown in Table IV. The set of predictors accounted for a significant proportion of the variance in SIP-total (15.9%), PAIS-sex (20.9%) and ROF-total scores (16.4%) (all *P*-values ⩽ 0.001). Education was a significant predictor of all QOL indices with less educated patients exhibiting *poorer* status. Time since BMT and age at BMT were significant predictors of three of four QOL indices, the exception being POMS-TMD scores. Increased time since BMT and younger age at BMT were associated with better QOL status.

A parallel set of analyses was conducted for allogeneic recipients only ($n = 93$). A set of nine predictor variables was used, including gender, age at BMT, time since BMT, dose of TBI used in pre-BMT conditioning, education, disease status, marital status, presence of chronic GVHD and extent of marrow graft match. Results are displayed in Table V. The set of predictors accounted for a significant proportion of variance in SIP-total (27.2%), PAIS-sex (41.8%) and ROF-total scores (26.4%) (all *P*-values <0.01). Lower level of education was associated with *poorer* QOL for all four QOL indices. More advanced disease and shorter time since BMT were associated with *poorer* status for three of four QOL indices, the exception in both cases being POMS-TMD scores. Older age at BMT was significantly associated with poorer ROF-total and PAIS-sex scores.

*Chronic GVHD and QOL outcomes* The multivariate analyses indicated that chronic GVHD was associated only with poorer PAIS-sex scores. To explore this further, item scores on this subscale were contrasted using *t*-test analyses for individuals with or without chronic GVHD. A significant difference was obtained for only one of seven items: individuals with chronic GVHD reported more concern regarding decreases in attractiveness [$t$ (91) = 2.41, $P < 0.05$]. No differences were obtained for items assessing other aspects of sexual desire or activity. Chi-square comparison of those with and without chronic GVHD with regard to ROF reports of whether personal appearance and sexual activity were 'normal' mirrored these results: individuals with chronic GVHD were more likely to report their appearance was 'not normal' relative to those without [28% *vs* 4%, $X^2$ (2) = 11.13, $P < 0.01$]. No differences in regard to whether the ability to engage in sexual activity had returned to normal were found.

### Discussion

The present study confirms findings from previous research, reports new findings and suggest avenues for future research. Consistent with previous studies, some BMT recipients reported excellent QOL. However, most recipients reported their physical health and QOL to be compromised to a degree. For example, BMT recipients perceived their current health and QOL to be poorer than that of a typical person their age as well as poorer than their own prediagnosis health and QOL. These deficits cannot be attributed solely to BMT, however, since the initial diagnosis and treatment of the original underlying malignant disease can have a significant negative impact in itself (Syrjala *et al.*, 1993). Regardless of the cause of post-BMT QOL deficits, it is significant that, even when BMT has been 'curative', most BMT recipients do not view themselves as having recovered their premorbid health and QOL.

Also confirming prior research (Lesko, 1993; Andrykowski, 1994), lower level of education and older age at BMT

**Table IV** Beta weights for multiple regression analysis of principal QOL variables for entire sample

| | Dependent variable | | | |
|---|---|---|---|---|
| *Predictor variable* | *POMS-TMD* | *SIP-total* | *ROF-total* | *PAIS-sex* |
| Gender[a] | 0.07 | 0.08 | 0.09 | 0.29*** |
| Type of BMT[b] | 0.05 | 0.12 | 0.16* | 0.17* |
| Time post BMT | 0.06 | −0.21** | −0.23** | −0.21* |
| Education | −0.19* | −0.25*** | −0.22** | −0.14* |
| Age at BMT | 0.07 | 0.21** | 0.21** | 0.28** |
| Dose of TBI | 0.04 | 0.07 | 0.08 | 0.07 |
| Disease status at BMT[c] | 0.07 | 0.13* | 0.13* | 0.09 |
| Marital status[d] | 0.08 | −0.03 | 0.04 | −0.01 |
| Multiple *R* | 0.256 | 0.398 | 0.405 | 0.457 |
| Variance accounted for | 6.5% | 15.9% | 16.4% | 20.9% |
| *F*-value[e] | 1.65 | 4.50*** | 4.69*** | 5.65*** |

Note: High scores mean poorer status for all variables. ***$P < 0.001$; **$P < 0.01$; *$P < 0.05$. [a]Coded as male = 0; female = 1. [b]Coded as autologous = 0; allogeneic = 1. [c]Coded as 1 = first remission; first relapse or first chronic phase CML; 2 = all others. [d]Coded as unmarried = 1; married = 2. [e]d.f. = 8, 191 except for PAIS-sexual relationships where d.f. = 8, 171.

**Table V** Beta weights for multiple regression analysis of principal QOL variables for allogeneic BMT recipients

| | Dependent variable | | | |
|---|---|---|---|---|
| *Predictor variable* | *POMS-TMD* | *SIP-total* | *ROF-total* | *PAIS-sex* |
| Gender[a] | 0.05 | −0.09 | −0.02 | 0.34*** |
| Marrow graft match[b] | −0.13 | −0.07 | 0.06 | 0.01 |
| Time post BMT | −0.02 | −0.27* | −0.29** | −0.31** |
| Education | −0.25* | −0.34** | −0.23* | −0.21* |
| Age at BMT | 0.12 | 0.21 | 0.36** | 0.48*** |
| Dose of TBI | −0.11 | −0.09 | 0.08 | −0.03 |
| Disease status at BMT[c] | 0.19 | 0.25* | 0.21* | 0.27* |
| Chronic GVHD[d] | 0.08 | 0.05 | 0.17 | 0.22* |
| Marital status[e] | −0.06 | −0.13 | −0.09 | −0.22* |
| Multiple *R* | 0.374 | 0.521 | 0.514 | 0.647 |
| Variance accounted for | 14.0% | 27.2% | 26.4% | 41.8% |
| *F*-value[f] | 1.46 | 3.44** | 3.32** | 5.76*** |

Note: High scores mean poorer status for all variables. ***$P < 0.001$; **$P < 0.01$; *$P < 0.05$. [a]Coded as male = 0; female = 1. [b]Coded as histocompatible = 0; haploidentical or MUD = 1. [c]Coded as 1 = first remission; first relapse or first chronic phase CML; 2 = all others. [d]Coded as no = 0; yes = 1. [e]Coded as unmarried = 1; married = 2. [f]d.f. = 9, 83 except for PAIS-sexual relationships where d.f. = 9, 72.

were consistent 'risk factors' for poorer QOL. The link between education and QOL might reflect better access to financial, medical or psychological resources which can facilitate post-BMT adjustment (Hobfall, 1989). The link between age and QOL could reflect age-related differences evident in the general population. However, patients rated their physical health and QOL to be poorer than that of a typical person their age, suggesting that this explanation is inadequate. More likely, older recipients are less able to tolerate the physical rigours imposed by conventional cytotoxic therapy and or BMT, thus compromising recovery of physical and functional status.

Finally, our data support previous research suggesting that chronic GVHD is not associated with post-BMT QOL (Andrykowski *et al.*, 1990). Because of the wide use of GVHD prophylaxis, GVHD was generally mild, thus limiting its potential impact on QOL. Chronic GVHD was associated only with poorer sexual relationship functioning in allogeneic recipients and was primarily due to the greater concern regarding personal appearance among those with chronic GVHD.

New findings from the present study include observations that post-BMT QOL is associated to some degree with disease status at BMT, type of BMT and time since BMT. Poorer QOL was associated with more advanced disease at BMT. This was particularly apparent for indices of functional status (SIP-total, ROF-total) and for allogeneic recipients. Since this variable has not been examined in previous studies of post-BMT QOL, replication is warranted. However, this finding suggests that physical and functional recovery may be inversely associated with extent of pre-BMT cytotoxic treatment. Relative to autologous recipients, allogeneic recipients reported poorer QOL. Allogeneic recipients reported: (a) less 'normal' functioning on the ROF, (b) more physical symptoms, (c) poorer sexual relationship functioning and (d) more unemployment. While reasons for the poorer QOL of allogeneic recipients cannot be unambigously determined in the absence of a population-based (i.e. single disease) prospective study, our findings are helpful from both clinical and policy perspectives. To the degree that our sample is representative of the allogeneic and autologous BMT experience, our data suggest that, in general, autologous BMT is associated with fewer post-BMT QOL deficits. This information could be helpful in the process of obtaining informed consent as well as planning for delivery of clinical rehabilitation services. Finally, a consistent, positive relationship between time post BMT and QOL indices of functional status was obtained. In contrast to previous research (Wolcott *et al.*, 1986; Andrykowski *et al.*, 1989; Belec, 1992) our data suggest that functional status might improve across time following the first year post BMT. Prospective, longitudinal research will be necessary to establish more firmly the temporary trajectory of QOL outcomes. Establishment of the type and timing of specific QOL deficits is critical to the timing of rehabilitation efforts to promote post-BMT QOL.

In addition to identifying variables associated with post-BMT QOL, our study identified variables which were consistently *not* associated with post-BMT QOL. These included differences in underlying diagnosis, dose of TBI and, for allogeneic recipients, extent of marrow graft match and type of GVHD prophylaxis. Analyses of the relationships between QOL and disease diagnosis, extent of marrow graft match and GVHD prophylaxis are unique to this study and thus merit replication. However, our finding of a lack of association between TBI dose and QOL contrasts with previous research (Andrykowski *et al.*, 1990). This difference could be due to study differences in case mix, indices used to assess QOL or other variables included in the regression model. Owing to the larger scope and use of a more comprehensive regression model in the present study, one is tempted to conclude that TBI might have less impact upon QOL than previously suggested.

Our data suggest several specific domains where post-BMT QOL is particularly likely to be compromised. These include fatigue, occupational disability, sleep difficulties and sexual relationships and functioning. Consistent with previous investigations (Andrykowski, 1990; Belec, 1992; Mumma *et al.*, 1992), difficulties with regard to reduced weakness, fatigue and the ability to engage in vigorous activities were pronounced. Clearly, more detailed study of fatigue in adult BMT recipients is warranted. Also consistent with previous research (Andrykowski *et al.*, 1990; Wingard *et al.*, 1991; Syrjala *et al.*, 1993), evidence for employment difficulties among BMT recipients was found. A third of our sample were unemployed or retired at the time of participation and cited health difficulties as the cause of such. While loss of strength and stamina contributed to unemployment in some instances, other potential causes include discrimination directed at cancer survivors (Hoffman, 1991), concerns over infection, hesitancy regarding relinquishment of the 'sick role' (Mechanic and Volkart, 1961), or simply that the benefits of being unemployed exceed those of being employed. Unemployment among BMT survivors is a concern because of both the financial burdens borne by many BMT patients and their families as well as the link between unemployment and pyschosocial distress (Dew *et al.*, 1991). Sleep difficulties also emerged as a common complaint. 'Sleep problems' was the second most frequently reported symptom on the SER. Since research has not identified sleep difficulties as a significant problem, further investigation of the aetiology and impact of sleep difficulties in BMT recipients is warranted, particularly in view of its potential contribution to fatigue in BMT patients.

Finally, reports of difficulties with sexual relationships and functioning were common, confirming previous investigations (Baruch *et al.*, 1991; Chao *et al.*, 1992; Mumma *et al.*, 1992; Vose *et al.*, 1992; Wingard *et al.*, 1994). Comparison of PAIS-sex scores in our sample with cancer patient norms (Derogatis and Lopez, 1983) indicated that 22 BMT recipients (12%) scored at least one standard deviation above the normative mean. Thus, about 12% of BMT recipients can be considered to be experiencing serious sexual relationship dysfunction relative to other cancer patients. Given that sexual dysfunction is more common in cancer patients relative to the general population (Andersen, 1985), the number of BMT patients experiencing serious sexual relationship dysfucntion probably exceeds 12%. Such dysfunction is probably multifactorial in origin. Cytotoxic chemotherapy is known to affect gonadal function in both sexes (Sherins and Mulvihill, 1989) and can have a marked impact upon sexual response in females (Lesko, 1993). Ovarian failure is a common sequela of cytotoxic chemotherapy, resulting in symptoms of vaginal dryness, dysparaeunia, decreased libido and vaginal epithelium atrophy (Sanders *et al.*, 1988, 1989; Cust *et al.*, 1989; Schubert *et al.*, 1990). While risk for post-chemotherapy ovarian failure increases with age, even younger women may exhibit transient symptoms of ovarian dysfunction for 2–6 years post BMT (Sanders *et al.*, 1988). Clearly, sexual difficulties are common in BMT recipients and require monitoring, particularly in women. Further study of the aetiology of post-BMT sexual dysfunction as well as development and implementation of clinical interventions, such as hormone replacement therapy, is warranted (Ostroff and Lesko, 1991; Lesko, 1993).

### *Strengths and limitations of the present study*

This is the largest and most comprehensive study of post-BMT QOL to date. All other large-scale studies of post-BMT QOL have been single-institution studies with findings necessarily circumscribed by the patients, diagnoses and treatment protocols represented in the case mix at that institution. The unique, multicentre collaboration involved in this study greatly increased the generalisability of our findings. In addition, this collaboration allowed us to accumulate a sample of sufficient size and heterogeneity to allow study of relationships between QOL and heretofore unexamined variables such as diagnosis and disease status at BMT, type of GVHD prophylaxis or type of BMT.

On the other hand, several weaknesses exist in the present study. First, a prospective, longitudinal design would have been preferable to the cross-sectional design we employed. Few institutions, however, have the patient census necessary to complete such an effort within a reasonable time frame. Collaborative efforts, like the present study or, even better, inclusion of QOL assessments in cooperative clinical trials (Nayfield *et al*., 1992) will be necessary to advance the field. Second, the instruments that we utilised to assess QOL have not been validated for use with BMT recipients. However, most have been validated for use with cancer patients and, in part, this is why we limited our study sample to BMT recipients with malignant disease. Third, owing to the number of data available, multiple tests of significance were required, thus increasing the likelihood of type I error. While significant findings should always be viewed with caution under such circumstances, we believe the fact that many of our analyses were driven by a priori hypotheses and/or confirmed previous findings increases confidence in our results. Finally, while statistically significant results emerged for many analyses, the clinical significance of our findings is unclear. Given the subjective nature of QOL (Aaronson, 1989), it is difficult to identify when statistical differences between groups are clinically important. For example, difficulties with fatigue, sexual functioning or employment will vary in clinical significance as a function of individuals' identities, circumstances and goals. Our data indicate common post-BMT problem areas and their associated risk factors. This can serve to focus clinical attention with intervention efforts mobilised if deemed clinically appropriate.

### *Conclusion*

Maximisation of post-BMT QOL requires rehabilitation services targeted for specific areas of difficulty in specific recipients at specific times during post-BMT recovery. Past research has done well in identifying areas where QOL might be compromised following BMT, but more in-depth studies of specific areas of difficulty, for example sexual functioning, underemployment, fatigue or sleep disturbance, would be useful. In contrast, less progress has been made in identifying specific BMT patients at risk for post-BMT difficulties, and still less progress has been made regarding identification of critical periods following BMT when particular difficulties might be manifest. Our data suggest that only a minority of variation in post-BMT QOL can be accounted for by demographic, disease and treatment variables. Understanding of variation in post-BMT QOL will require consideration of variables such as social support, coping style, psychiatric history or pre-BMT expectations, and longitudinal research will be needed to map the temporal trajectory of post-BMT QOL and suggest critical periods when QOL might be compromised.

**Acknowledgements**
This research was supported by research grants 1 RO1 CA49431-01A1 and PO1 CA39542 from the National Cancer Institute, and by American Cancer Society Junior Faculty Research Award No. JFRA-387 to the senior author. We would like to thank Jean Sunega MS for assistance in data collection and data entry. We would also like to thank all of the patients who so graciously participated in this research.

### References

AARONSON NK. (1989). Quality of life assessment in clinical trials: methodologic issues. *Controlled Clin. Trials*, **10**, 195S-208S.

ANDERSEN BL. (1985). Sexual functioning morbidity among cancer survivors: current status and future research directions. *Cancer*, **55**, 1835-1842.

ANDRYKOWSKI MA. (1994). Psychosocial factors in bone marrow transplantation: a review and recommendations for research. *Bone Marrow Transplant*, **13**, 357-375.

ANDRYKOWSKI MA, HENSLEE PJ AND BARNETT RL. (1989). Longitudinal assessment of psychosocial functioning of adult survivors of allogeneic bone marrow transplantation. *Bone Marrow Transplant.*, **4**, 505-09.

ANDRYKOWSKI MA, ALTMAIER EM, BARNETT RL, OTIS ML, GINGRICH R AND HENSLEE-DOWNEY PJ. (1990). Quality of life in adult survivors of allogeneic bone marrow transplantation: correlates and comparison with matched renal transplant recipients. *Transplantation*, **52**, 399-406.

ANDRYKOWSKI MA, BRADY MJ AND HUNT JW. (1993). Positive psychosocial adjustment in potential bone marrow transplant recipients: cancer as a psychosocial transition. *Psycho-Oncology*., **2**, 261-276.

ANDRYKOWSKI MA, BRADY MJ, GREINER CB, ALTMAIER EM, BURISH TG, ANTIN JH, GINGRICH R, MCGARIGLE C AND HENSLEE-DOWNEY PJ. (1995). 'Returning to Normal' following bone marrow transplantation: outcomes, expectations, and informed consent. *Bone Marrow Transplant* (in press).

BAKER F, CURBOW B AND WINGARD JR. (1991). Role retention and quality of life of bone marrow transplant survivors. *Soc. Sci. Med.*, **32**, 697-704.

BAKER F, WINGARD JR, CURBOW B, ZABORA J, JODREY D, FOGARTY L AND LEGRO M. (1994). Quality of life of bone marrow transplant long-term survivors. *Bone Marrow Transplant.*, **13**, 589-596.

BARUCH J, BENJAMIN S, TRELEAVEN J, WILCOX AH, BARRON JL AND POWLES R. (1991). Male sexual dysfunction following bone marrow transplantation. *Bone Marrow Transplant.*, **2** (Suppl. 7), 52.

BELEC RH. (1992). Quality of Life: Perceptions of long-term survivors of bone marrow transplantation. *Oncol. Nurs. Forum.*, **19**, 31-7.

BERGNER M, BOBBITT RA, CARTER WB AND GILSON BS. (1981). The sickness impact profile: development and final revision of a health status measure. *Med. Care*, **19**, 787-805.

BORTIN MM AND RIMM AA. (1989). Increasing utilization of bone marrow transplantation: II. Results of the 1985-1987 survey. *Transplantation*, **48**, 453-8.

CANTRIL H. (1965). *The Patterns of Human Concern*. Rutgers University Press: New Brunswick, NJ.

CHAMPLIN RE AND GALE RP. (1984). Role of bone marrow transplantation in the treatment of hematologic malignancies and solid tumours: critical review of syngeneic, autologous, and allogeneic transplants. *Cancer Treat. Rep.*, **68**, 145-61.

CHAO NJ AND BLUME KG. (1989). Bone marrow transplantation. Part I. Allogeneic. *West. J. Med.*, **151**, 638-43.

CHAO NJ AND BLUME KG. (1990). Bone marrow transplantation. Part II. Autologous. *West. J. Med.*, **152**, 46-51.

CHAO NJ, TIERNEY DK, BLOOM JR, LONG GD, BARR TA, STALLBAUM BA, WONG RM, NEGRIN RS, HORNING SJ AND BLUME KG. (1992). Dynamic assessment of quality of life after autologous bone marrow transplantation. *Blood*, **80**, 825-30.

CUST MP, WHITEHEAD MI, POWLES R, HUNTER M AND MILLIKEN S. (1989). Consequences and treatment of ovarian failure after total body irradiation for leukaemia. *Br. Med. J.*, **299**, 1494-97.

DEEG HJ. (1990). Delayed complications and long-term effects after bone marrow transplantations. *Hematol. Oncol. Clin. N. Am.*, **4**, 641-57.

DEROGATIS LR. (1986). The Psychosocial Adjustment to Illness Scale (PAIS). *J. Psychosom. Res.*, **30**, 77-91.

DEROGATIS LR AND LOPEZ M. (1983). PAIS & PAIS-SR: Administration, Scoring and Procedures Manual, Vol. I. Clinical Psychometric Research: Baltimore, MD.

DEW MA, PENKOWER L AND BROMET EJ. (1991). Effects of unemployment on mental health in the contemporary family. *Behav. Modif.*, **15**, 501-44.

HOBFALL SE. (1989). Conservation of resources: a new attempt at conceptualizing stress. *Am. Psychol.*, **44**, 513-524.

HOFFMAN B. (1991). Employment discrimination: another hurdle for cancer survivors. *Cancer Invest.*, **9**, 589-95.

KOLB HJ AND BENDER-GOTZE C. (1990). Late complications after allogeneic bone marrow transplantation for leukaemia. *Bone Marrow Transplant.*, **6**, 61-72.

LESKO LM. (1993). Psychiatric aspects of bone marrow transplantation: Part II: Life beyond transplant. *Psycho-Oncology*., **2**, 185-193.

McNair PM, Lorr M and Droppelman L. (1981). *POMS Manual*, 2nd edn. Educational and Industrial Testing Service: Dan Diego, CA.

Mechanic D and Volkart EH. (1961). Stress, illness behavior, and the sick role. *Am. Sociol. Rev.*, **26**, 51-8.

Moinpour CM, Feigl P, Metch B, Hayden KA, Meyskens FL and Crowley J. (1989). Quality of life end points in cancer clinical trials: review and recommendations. *J. Natl Cancer Inst.*, **81**, 485-95.

Mumma GH, Mashberg D and Lesko LM. (1992). Long-term psychosexual adjustment of acute leukemia survivors: impact of marrow transplantation versus conventional chemotherapy. *Gen. Hosp. Psychiatry*, **14**, 43-55.

Nayfield SG, Ganz PA, Moinpour CM, Cella DF and Hailey BJ. (1992). Report from a National Cancer Institute (USA) workshop on quality of life assessment in cancer clinical trials. *Qual. Life Res.*, **1**, 203-10.

Ostroff JS and Lesko LM. (1991). Psychosexual adjustment and fertility issues. In *Bone Marrow Transplantation: Principles, Practices and Nursing Insights*, Whedon MB. (ed.) pp. 313-333. Jones & Bartlett Publishers: Boston.

Sanders JE, Buckner CD, Amos D, Levy W, Appelbaum FR, Doney K, Storb R, Sullivan KM, Witherspoon RP and Thomas ED. (1988). Ovarian function following marrow transplantation for aplastic anemia or leukemia. *J. Clin. Oncol.*, **6**, 813-18.

Sanders JE, Sullivan KM, Witherspoon R, Doney K, Anasetti C, Beatty P and Petersen FB. (1989). Long term effects and quality of life in children and adults after bone marrow transplantation. *Bone Marrow Transplant.*, **4**, 27-29.

Schmidt GM, Niland JC, Forman SJ, Fonbuena PP, Dagis AC, Grant MM, Ferrell BR, Barr TA, Stallbaum BA, Chao NJ and Blume KG. (1993). Extended follow-up in 212 long-term allogeneic bone marrow transplant survivors: issues of quality of life. *Transplantation*, **55**, 551-557.

Schubert MA, Sullivan KM, Schubert JN, Hansen M, Sanders JE, O'Quigley J, Witherspoon RP, Buckner CD and Thomas ED. (1990). Gynecological abnormalities following allogeneic bone marrow transplantation. *Bone Marrow Transplant.*, **5**, 425-430.

Sherins RJ and Mulvihill JJ. (1989). Gonadal dysfunction. In *Cancer: Principles and Practice of Oncology*, Devita VT, Helman S and Rosenberg SA. (eds) pp. 2170-2181. Lippincott: Philadelphia.

Siegel S. (1956). *Nonparametric Statistics for the Behavioral Sciences*. McGraw Hill: New York.

Syrjala KL, Chapko MK, Vitaliano PP, Cummings C and Sullivan KM. (1993). Recovery after allogeneic marrow transplantation: prospective study of predictors of long-term physical and psychosocial functioning. *Bone Marrow Transplant.*, **11**, 319-327.

Vose JM, Kennedy BC, Bierman PJ, Kessinger A and Armitage JO. (1992). Long-term sequelae of autologous bone marrow or peripheral stem cell transplantation for lymphoid malignancies. *Cancer*, **69**, 784-89.

Ware JE. (1984). Conceptualizing disease impact and treatment outcomes. *Cancer*, **53**, 2316-23.

Wingard JR, Curbow B, Baker F and Piantadosi S. (1991). Health functional status and employment of adult survivors of bone marrow transplantation. *Ann. Intern. Med.*, **114**, 113-118.

Wingard JR, Curbow B, Baker F, Zabora J and Piantadosi S. (1994). Sexual satisfaction in survivors of bone marrow transplantation. *Bone Marrow Transplant.*, **9**, 185-90.

Wolcott DL, Wellisch DK, Fawzy FI and Landsuerk J. (1986). Adaptation of adult bone marrow transplant recipient long-term survivors. *Transplant*, **41**, 478-484.